# The Burden of Critical Illness in Hospitalized Children in Low- and Middle-Income Countries: Protocol for a Systematic Review and Meta-Analysis

**DOI:** 10.3389/fped.2022.756643

**Published:** 2022-03-16

**Authors:** Teresa B. Kortz, Katie R. Nielsen, Rishi P. Mediratta, Hailey Reeves, Nicole F. O'Brien, Jan Hau Lee, Jonah E. Attebery, Emaan G. Bhutta, Carter Biewen, Alvaro Coronado Munoz, Mary L. deAlmeida, Yudy Fonseca, Shubhada Hooli, Hunter Johnson, Niranjan Kissoon, Mara L. Leimanis-Laurens, Amanda M. McCarthy, Carol Pineda, Kenneth E. Remy, Sara C. Sanders, Yemisi Takwoingi, Matthew O. Wiens, Adnan T. Bhutta

**Affiliations:** ^1^Division of Critical Care, Department of Pediatrics, University of California, San Francisco, San Francisco, CA, United States; ^2^Institute for Global Health Sciences, University of California, San Francisco, San Francisco, CA, United States; ^3^Division of Critical Care, Department of Pediatrics, University of Washington, Seattle, WA, United States; ^4^Department of Global Health, University of Washington, Seattle, WA, United States; ^5^Division of Pediatric Hospital Medicine, Department of Pediatrics, Stanford University School of Medicine, Stanford, CA, United States; ^6^Division of Critical Care Medicine, Department of Pediatrics, The Ohio State University/Nationwide Children's Hospital, Columbus, OH, United States; ^7^Children's Intensive Care Unit, KK Women's and Children's Hospital, Singapore, Singapore; ^8^SingHealth Duke-NUS Global Health Institute, Singapore, Singapore; ^9^Division of Critical Care, Department of Pediatrics, University of Colorado, Aurora, CO, United States; ^10^Eberly College of Science, Pennsylvania State University, State College, PA, United States; ^11^Division of Critical Care, Department of Pediatrics, University of Texas Health Science Center at Houston, Houston, TX, United States; ^12^Division of Critical Care, Department of Pediatrics, Emory University, Atlanta, GA, United States; ^13^Division of Pediatric Critical Care Medicine, Department of Pediatrics, University of Maryland School of Medicine, Baltimore, MD, United States; ^14^Section of Pediatric Emergency Medicine, Department of Pediatrics, Baylor College of Medicine, Houston, TX, United States; ^15^Children's and Women's Global Health, University of British Columbia, Vancouver, BC, Canada; ^16^Department of Pediatrics and Emergency Medicine, University of British Columbia, Vancouver, BC, Canada; ^17^Pediatric Critical Care Unit, Helen DeVos Children's Hospital, Grand Rapids, MI, United States; ^18^Department of Pediatrics and Human Development, College of Human Medicine, Michigan State University, East Lansing, MI, United States; ^19^Division of Critical Care, Department of Pediatrics, Baystate Medical Center, University of Massachusetts Chan Medical School, Springfield, MA, United States; ^20^Division of Critical Care Medicine, Department of Pediatrics, Washington University School of Medicine, St. Louis, MO, United States; ^21^Division of Pediatric Critical Care Medicine, Department of Pediatrics, Rainbow Babies and Children's Hospital, Case Western Reserve University, Cleveland, OH, United States; ^22^Test Evaluation Research Group, Institute of Applied Health Research, University of Birmingham, Birmingham, United Kingdom; ^23^National Institute for Health Research Birmingham Biomedical Research Centre, University Hospitals Birmingham National Health Service Foundation Trust and University of Birmingham, Birmingham, United Kingdom; ^24^Department of Anesthesiology, Pharmacology and Therapeutics, University of British Columbia, Vancouver, BC, Canada; ^25^Mbarara University of Science and Technology, Mbarara, Uganda

**Keywords:** critical illness, resource limited setting, pediatrics—children, child health, global health, hospitalization, low- and middle-income countries (LMIC)

## Abstract

**Background:**

The majority of childhood deaths occur in low- and middle-income countries (LMICs). Many of these deaths are avoidable with basic critical care interventions. Quantifying the burden of pediatric critical illness in LMICs is essential for targeting interventions to reduce childhood mortality.

**Objective:**

To determine the burden of hospitalization and mortality associated with acute pediatric critical illness in LMICs through a systematic review and meta-analysis of the literature.

**Data Sources and Search Strategy:**

We will identify eligible studies by searching MEDLINE, EMBASE, CINAHL, and LILACS using MeSH terms and keywords. Results will be limited to infants or children (ages >28 days to 12 years) hospitalized in LMICs and publications in English, Spanish, or French. Publications with non-original data (e.g., comments, editorials, letters, notes, conference materials) will be excluded.

**Study Selection:**

We will include observational studies published since January 1, 2005, that meet all eligibility criteria and for which a full text can be located.

**Data Extraction:**

Data extraction will include information related to study characteristics, hospital characteristics, underlying population characteristics, patient population characteristics, and outcomes.

**Data Synthesis:**

We will extract and report data on study, hospital, and patient characteristics; outcomes; and risk of bias. We will report the causes of admission and mortality by region, country income level, and age. We will report or calculate the case fatality rate (CFR) for each diagnosis when data allow.

**Conclusions:**

By understanding the burden of pediatric critical illness in LMICs, we can advocate for resources and inform resource allocation and investment decisions to improve the management and outcomes of children with acute pediatric critical illness in LMICs.

## Introduction

Greater than 80% of the global 6.64 million annual deaths in children and adolescents in 2017 occurred in low- and middle-income countries (LMICs) (1). Acute pediatric illnesses (e.g., sepsis, pneumonia, diarrheal disease, trauma) are the leading causes of death and disability outside of the neonatal period (1–5). The World Health Organization defines acute pediatric critical illness as “any severe problem with the airway, breathing, or circulation, or acute deterioration of conscious state; [which] includes apnea, upper airway obstruction, hypoxemia, central cyanosis, severe respiratory distress, total inability to feed, shock, severe dehydration, active bleeding requiring transfusion, unconsciousness, or seizures” (6). A significant number of children's lives could be saved with supportive critical care interventions, such as fluid resuscitation, high-flow oxygen therapy, non-invasive and invasive mechanical ventilation, and vasoactive support (7–11). Unfortunately, critical care services, defined as hospital care for children with sudden, serious reversible disease, are not universally available and are frequently lacking in LMIC settings, where disease burden, both in terms of hospitalization and mortality, is the highest (7). Furthermore, it is difficult to assess the burden of critical illness in settings without formal critical care services, where critical illness is frequently managed in emergency departments and inpatient wards.

Several recent global point prevalence studies have described the prevalence of key, individual acute pediatric critical illnesses. The Pediatric Acute Lung Injury Ventilation (PALIVE) study, conducted in 59 pediatric intensive care units (PICUs), found that 10.8% of children were diagnosed with acute lung injury (12). The Pediatric Acute Respiratory Distress Syndrome Incidence and Epidemiology (PARDIE) study reported a prevalence of pediatric acute respiratory distress syndrome of 3.2% and an associated mortality of 17% mortality in children admitted to 145 PICUs from 27 countries (13). The International Survey of Critically Ill Children with Acute Neurological Insults (PANGEA) study conducted in 107 PICUs across 23 countries found an overall prevalence of acute neurologic insult to be 16.2% and all-cause hospital mortality was 12% (14). Finally, the Sepsis Prevalence, Outcomes, and Therapies (SPROUT) study was conducted in 128 PICUs across 26 countries and demonstrated a prevalence of pediatric severe sepsis of 8.2% with a hospital mortality of 25% (15).

While each of these studies contributed significant knowledge about specific acute pediatric critical illnesses, there are limitations to the available data. The first limitation stems from the focus on a single, critical illness or insult as opposed to all pediatric critical illnesses. There is substantial overlap between illnesses (e.g., pneumonia is a frequent cause of sepsis). In addition, critical care resources support patients with many diagnoses (e.g., mechanical ventilation supports children with pneumonia, shock, or trauma), and resource availability, or lack thereof, greatly impacts patient outcomes. A narrow, illness-specific view fails to capture the burden of pediatric critical illness, which makes it difficult to prioritize resources and achieve the greatest potential impact on child mortality. The most significant limitation, however, is that current global pediatric critical illness point prevalence studies do not reflect the prevalence of disease is LMICs. The PALIVE study was conducted exclusively in North American and European countries; (12) no low-income countries were included in the PARDIE study; (13) approximately 80% of PANGEA study sites were in North America and Europe; (14) and the SPROUT study, while it included several LMICs, did not include any countries from sub-Saharan Africa outside of South Africa (15). Each of these global point prevalence studies required PICU admission as an inclusion criterion. This drastically limited which centers and settings could participate and may have resulted in a gross underestimation of pediatric critical illness in LMICs where critical illness may be managed in sites without a formal PICU (7).

In this systematic review, we will describe the burden of hospitalizations and mortality associated with acute pediatric critical illness in LMICs including in settings that may not have a PICU or formal intensive care services. This review will contribute to our knowledge of the etiologies and prevalence of acute pediatric critical illness in settings with the highest burden of disease. This information will help guide decisions justifying resource allocation and investment as well as inform educational, policy, and research priorities to improve outcomes following acute pediatric critical illness globally.

## Materials and Methods

### Objectives

The objectives of this study are to (1) determine common causes of pediatric hospital admissions (critical and non-critical) and mortality in LMICs; (2) determine the prevalence of and mortality associated with acute pediatric critical illness in LMICs; and (3) analyze the differences in common causes of critical illness by age and region.

### Protocol and Registration

This protocol follows the Preferred Reporting Items for Systematic Review and Meta-Analysis Protocols guidelines (PRISMA), is registered in the international prospective register of systematic reviews (PROSPERO #230228) and was organized and reviewed by the Pediatric Acute Lung Injury and Sepsis Investigator (PALISI) Network Global Health subgroup and PALISI Network Scientific committee (16, 17). The multinational and multidisciplinary scientific Working Group responsible for development of the systematic review protocol includes subject matter and/or methodology experts from across the globe who are members in good standing of the PALISI Global Health subgroup. The inclusion criteria are presented according to published guidelines for prevalence systematic reviews of observational studies (CoCoPop or condition, context, and population) (18).

### Population

The population of interest is a general pediatric admission population admitted to a hospital in a low-, lower-middle, or middle-income country (LMIC), defined below in Section Context. The age range of interest includes post-neonatal (>28 days of age) and pre-adolescent (<13 years of age) children; studies that do not include some portion of this age range (>28 days to <13 years) will be excluded. However, studies that include this age range (>28 days to <13 years) plus either neonates and/or adolescents will be included, if it is a pediatric study population (e.g., includes study participants <18 years of age). All hospital admissions, regardless of admission disposition (high-dependency unit, PICU, ward, etc.) will be included. Studies where the available denominator represents a specific patient population and not all hospital admissions, such as emergency department patients, neonatal intensive care admissions, pediatric intensive care admissions, and neonatal populations, will be excluded. In situations when the denominator of interest does not represent the entire general pediatric admission population due to study-imposed exclusions, Working Group members will assess these texts individually and decide whether the study exclusion criteria likely resulted in a significantly different case mix (i.e., highly prevalent condition, condition highly relevant to critical illness) compared to the overall, general pediatric admission population. If so, then the text will be excluded. If not, then it will be included and assessed for bias during quality assessment.

### Condition

The burden of critical illness is hospitalization or mortality due to a critical illness. Critical illness is defined as a state of ill health with vital organ system dysfunction and/or a high risk of imminent death. Studies must report the proportion of children with a specific admission diagnosis or cause of death (the numerator), such as pneumonia, human immunodeficiency virus (HIV), malaria, etc., relative to the number of general pediatric hospital admissions (the denominator) over that same period to be included. Both the numerator and denominator must represent the same patient population.

### Context

Observational studies (prospective or retrospective cohorts, surveillance studies, hospital database publications, cross-sectional studies, before data from before-and-after studies, registry data, etc.) must be published since January 1, 2005, in Spanish, French, or English to be included. For studies including data collected before the year of 2000, only data from 2000 to present will be included; however, if it is not possible to extract only data after the year 2000, the study will be excluded in its entirety. Exclusion of data before the year 2000 and the publication date of January 2005 were chosen to reflect recent trends in pediatric hospitalization and mortality.

Only studies conducted in LMICs will be included. Low- and middle-income country status will be determined by the Global Burden of Disease (GBD) 2017 Socio-Demographic Index (SDI) (19). The SDI is a composite indicator that includes indices of total fertility rate for women under age 25 years, mean education for people 15 years and older, and a lag-distributed income per capita. Socio-Demographic Index represents a country's overall development status and strongly correlates with health outcomes. Studies that present aggregated data representing multiple countries (e.g., multi-center study) will be included, and we will report regional data. Publications conducted in LMICs but not representative of the setting (e.g., medical mission, foreign military hospital, disaster response efforts) will be excluded.

Abstract only publications, case studies, narrative reviews, surveys, study protocols, comments, editorials, letters, notes, conference materials, interventional trials, and texts for which we cannot locate the full text will be excluded. The search may be updated prior to publication to include more recent publications.

### Data Sources and Search Strategy

**Figure 1:**
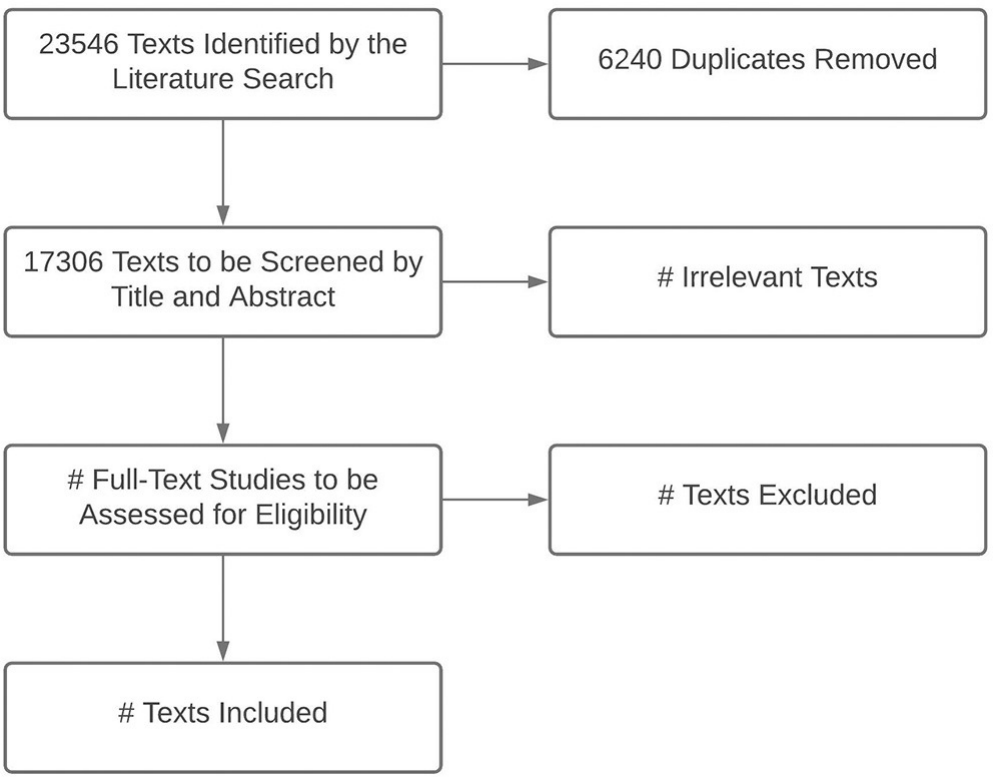
Preferred Reporting Items for Systematic Review and Meta-Analysis Protocols (PRISMA) flowchart for title and abstract screening and text selection from the final search (conducted March 1, 2021).

A search strategy was developed among co-investigators and an academic librarian and tested for feasibility. The final search results are shown in Figure 1. We identified eligible studies by searching Ovid MEDLINE (1946 to February 26, 2021, with Epub Ahead of Print, In-Process, and Other Non-Indexed Citations), EMBASE.com (1974 to March 2021), CINAHL (1981 to March 2021), and LILACS (1982 to March 2021) (Table 1). The MEDLINE search was performed using MeSH and key words for “hospitalization,” “patient admission,” “patient readmission,” “hospital units,” “critical care,” “intensive care,” “mortality,” and “developing countries.” Countries determined to be LMICs by SDI criteria were listed individually to increase the specificity of the search. The MEDLINE strategy was adapted to search EMBASE, CINAHL, and LILACS. All results were limited to infants or children (ages 29 days to 12 years) and publication years 2005 to present. There were no language restrictions; texts in languages other than English, Spanish, or French will be manually excluded during screening. Specified publication types were excluded in MEDLINE and EMBASE (e.g., comments, editorials, letters, notes, conference materials) (Supplementary Table 1).

**Table 1:** Summary of searched databases and number of texts identified by the search strategy.

**Database**	**Dates included**	**Date** **searched**	**Number of** **texts identified**
Ovid MEDLINE (R) Epub Ahead of Print In-Process and other Non-Indexed Citations Daily and Versions (R)	1946 to February 26, 2021	3/1/2021	11,240
EMBASE.com	1974 to Present (includes Medline 1966 to Present)	3/1/2021	11,403
Cumulative Index to Nursing and Allied Health Literature (CINAHL)	1981 to Present	3/1/2021	3,878
Latin American and Caribbean Health Sciences Literature (LILACS)	1982 to Present	3/1/2021	1,453
**Total**			**27,974**

### Study Selection and Screening Process

**Figure 2:**
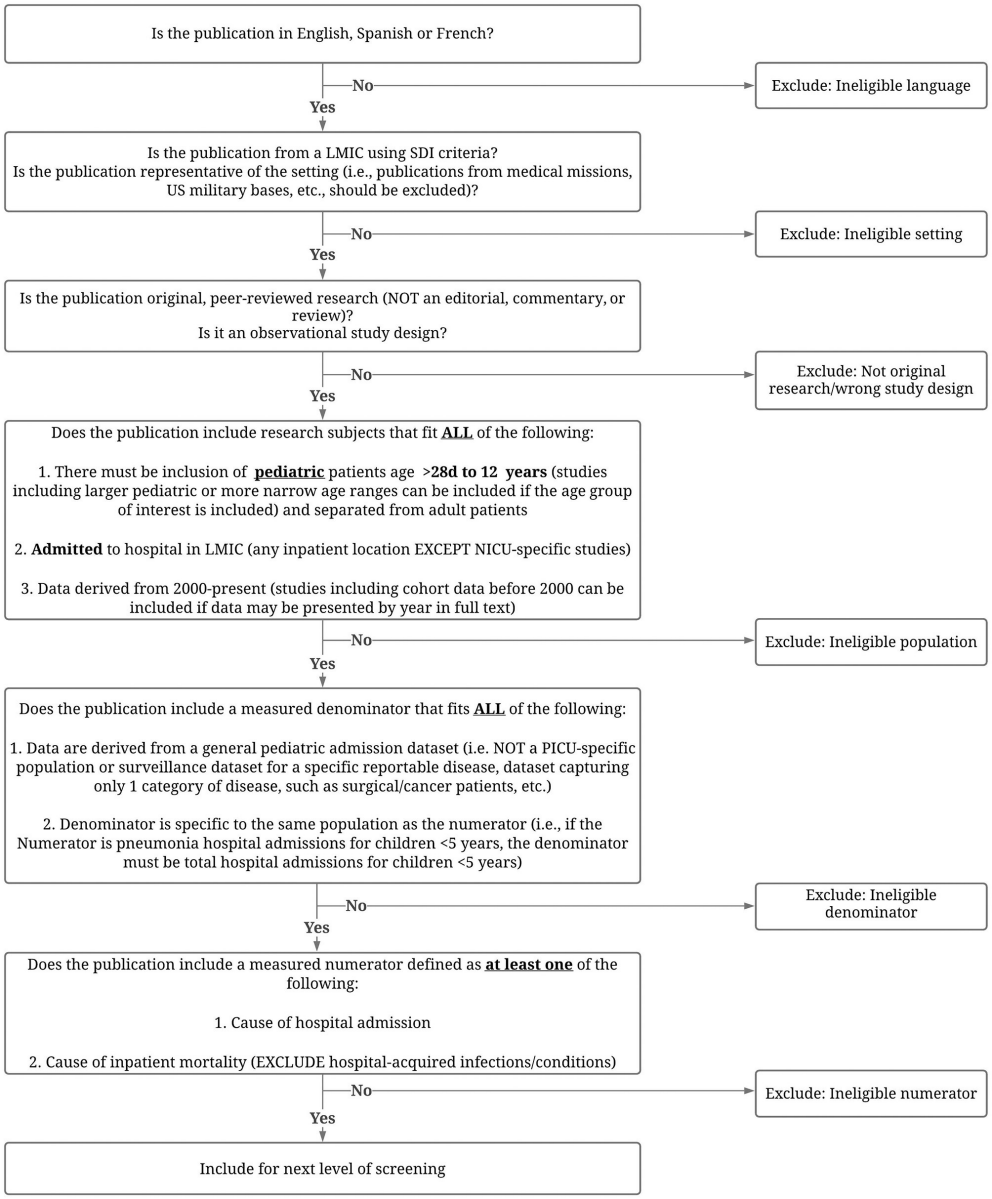
Approach to screening abstracts, titles and texts for eligibility. LMIC, low- and middle-income country; SDI, Socio-Demographic Index; NICU, neonatal intensive care unit; PICU, pediatric intensive care unit.

The titles from the search will be uploaded to and screened using Covidence (Veritas Health Innovation, Melbourne, Australia) (20). Covidence is a web-based systematic review platform designed to facilitate citation screening, full-text upload, and conflict resolution. Citations will be screened for eligibility based on title and abstract, and full text using a study-specific flowchart (Figure 2).

Working Group members will complete a training set of 25 citations (titles and abstracts) before initiating screening for the project. At least 5–10 true positives will be purposely included in the training set. The training set will be created by the study investigator (TK). Members of the Working Group will independently screen the titles and abstracts and then discuss and align on the final decision during a Working Group meeting.

Each title will be screened by two reviewers using the predetermined eligibility criteria. Specific Working Group Members fluent in non-English languages will be designated to review citations in Spanish (ACM, KN, TK, YF, CP) and French (ACM, HR, NO, CP). Titles that are eliminated by both reviewers will be rejected; titles accepted by both reviewers will advance to full-text screening; and titles in which a consensus is not reached will be resolved by a third member. Each full-text article will be assessed by two members of the Working Group for inclusion in the final set of articles for data extraction. At each screening and assessment phase, conflicts will be resolved by a third member of the Working Group using the conflict resolution function in Covidence.

For full texts with exclusion criteria, a reason for exclusion will be recorded (e.g., ineligible language; ineligible setting; not original research/wrong study design; ineligible population; ineligible denominator; ineligible numerator; full text not found; duplicate article). Texts identified by title and abstract screening will be excluded if the full text cannot be found after the following stepwise process is completed: search of available journal article subscriptions at two or more academic institutions; a general web-based search using Google; an Interlibrary Loan request from at least two academic institutions; an article request via direct email to the corresponding author or editor. For multiple publications from one dataset, we will only include the data once (e.g., the most recent or most relevant publication). For publications with multiple years of data presented by year or groups of years (e.g., vaccine surveillance studies), we will include the most recent year(s) as this is more likely to reflect the current epidemiology of disease. Publications with data from more than one country (e.g., global prevalence studies) will be considered for inclusion if either (a) all included countries meet LMIC criteria, or (b) data from LMICs can be extracted separately from non-LMIC data. We may contact authors to stratify data by age for already published texts.

### Data Extraction and Management

Data from all included full-text articles will be extracted by two, independent Working Group members and managed using REDCap, a secure, web-based application and electronic data capture tool hosted at the University of California, San Francisco (21). Data extraction conflicts will be resolved by a third member of the Working Group using the data comparison functionality in REDCap.

Data extraction will include information related to study characteristics (i.e., title, authors, year of publication, date of enrollment, urban/rural, country, language, journal, study design, sample size, inclusion/exclusion criteria, data source); hospital characteristics (public/private/faith-based, referral/district, community/academic, children's hospital, intensive care resources available, number of beds); underlying population characteristics (population served, proportion living in poverty, malaria rate, HIV rate, malnutrition rate); patient population characteristics (age, sex, presence of malnutrition, and other comorbidities); and outcomes (cause of admission, cause of death, length of stay).

### Risk of Bias in Individual Studies

Two members of the Working Group will independently assess the quality of each selected article and risk of bias using an adapted version of the Quality in Prognosis Studies (QUIPS) tool (22). While this review will not assess prognostic factors for admission and/or mortality, biases relevant to prognostic factors are similar to those relevant to the assessment of causes of these outcomes. The QUIPS tool includes six domains of bias, of which the three deemed appropriate for this review are: (1) study participation; (2) study attrition; and (3) prognostic factor (i.e., cause) measurement. The fourth, outcome measurement, is not relevant as this systematic review assesses causes of admission (and where relevant, death), and the cross-sectional nature precludes a temporally linked outcome to the cause. The fifth and sixth domains, study confounding and statistical analysis, respectively, were also deemed not relevant as the data to be extracted are counts. Issues around improper analyses will be adequately captured in domains 2 (attrition) and 3 (measurement of cause). The adapted domains, key issues, and items for consideration relevant to this review are shown in Table 2. Risk of bias will be classified as high, moderate, or low when the relationship between the listed causes and outcome is very likely to be, may be, or unlikely to be, respectively, different for participants and eligible non-participants. Conflicts in the risk of bias assessment will be resolved by discussion or by a member of the Working Group if consensus cannot be reached. We will produce one or more summary of findings tables that will provide an overview of the evidence to make the findings accessible to readers. The tables will include summaries of the methodological quality (risk of bias), precision of summary estimates (imprecision), concerns about heterogeneity (inconsistency), applicability of the findings to our review question (indirectness), and issues with publication bias. The tables will also include any additional limitations of the evidence. We will explore the impact of the risk of bias domains in sensitivity analyses.

**Table 2:** Risk of bias domains and questions adapted from the Quality in Prognosis Studies (QUIPS) criteria.

**Domain**	**Key Issue in this Review**	**Items for Consideration During Assessment**
Study participation	Do those subjects who are enrolled/analyzed represent the general admission population of this age group at this facility (or these facilities if multisite)?	(a) Adequate participation in the study by eligible persons (i.e., all those admitted in the target age group). (b) Description of the source population or population of interest. (c) Description of the baseline study sample. (d) Adequate description of the sampling frame and recruitment (i.e., if not a census sample, effort was made to ensure a representative sample of the admission population). (e) Adequate description of the period and place of recruitment (e.g., representative in terms of seasonality, natural fluctuations in causes based on time of day, day of week, etc.). (f) Adequate description of inclusion and exclusion criteria (i.e., any efforts in sample selection should be to make the sample more representative of the general admission population, not less).
Study attrition	Do those subjects who are enrolled represent those in whom the outcome (cause of admission, cause of death) is measured? This is especially relevant to those studies assessing both causes of admission AND causes of death.	(a) Those who are enrolled and those in whom a cause (of admission, death, etc.) was measured are the same. (b) Reasons for losses between enrollment (admission) and outcome ascertainment (cause of admission, cause of death) are provided. (c) Adequate description of participant losses. (d) There are no important differences between participants who completed the study and those who did not.
Listed causes of measurement (i.e., measurement of admission/death)	Do those subjects in whom a cause of admission/death is reported have this cause (or these causes) measured reliably?	(a) A clear definition or description of the listed causes is provided. (b) Method of the determination of causes valid and reliable. (c) The method and setting of measurement of listed causes is the same for all study participants. (d) Appropriate methods of imputation are used for missing listed causes data.

### Data Synthesis and Analysis

We will summarize data on study (author, publication year, study country, study design, sample size, ages included, data source), hospital [catchment population, type of hospital (level, affiliation, pediatric, etc.), number of health facility and pediatric beds, and available intensive care resources] and patient (median age, prevalence of comorbidities such as malnutrition, congenital heart disease, prematurity, malignancy, malaria, and anemia) characteristics; outcomes; and risk of bias assessment using tables, graphs, and narrative summaries. Continuous outcomes will be summarized using mean and standard deviations (SDs) or medians with interquartile ranges as appropriate. Binary outcomes will be summarized using frequencies and percentages.

The primary outcomes of interest are (1) cause of hospital admission and (2) cause of in-hospital mortality. Causes of hospital admissions will be further categorized as critical (potentially life-threatening) and non-critical (unlikely to be life threatening) based on group consensus and a review of the literature. If available, data for secondary outcomes will be collected including in-hospital mortality, case fatality rate (CFR), and length of hospital stay.

We will report the causes of admission and mortality (categorized by GBD grouping) by region (Central Europe, Eastern Europe, and Central Asia; Latin America and Caribbean; North Africa and Middle East; South Asia; Southeast Asia, East Asia, Oceania; Sub-Saharan Africa), SDI country income level (low-, lower-middle, or middle-income), and age (<5 years, 5–12 years).

When possible, we will report the CFR for each cause of admission and/or cause of death. This may require calculating these estimates from individual studies when not reported directly, provided that the necessary data to perform these calculations are reported. Causes of hospital admissions will be categorized as non-critical or critical (potentially life-threatening) by the same multinational, multidisciplinary scientific Working Group compiled of experts described above. The Working Group will reach consensus as to whether the reason for admission is consistent with vital organ system dysfunction and/or a high risk of imminent death based on a review of region-specific literature. We will explore different definitions and cut-offs for critical illness (proportion of total admissions, proportion of total mortality, CFR).

As the data allow, we will perform a meta-analysis on the proportions of causes of admission and causes of death, as well as the CFRs using random-effects models. We will conduct meta-regression, where possible, to explore predictors for all-cause and cause-specific mortality (pneumonia, sepsis, and diarrhea). Possible predictors will include SDI, facility type, and geographic region. Additionally, we will explore temporal trends in admission and mortality by age and region. We will consider subgroup analyses if we have adequate numbers of studies and/or patients within the included studies.

We will examine sources of heterogeneity, including differences in methodology, setting (urban vs. rural), region, income level, and patient populations (e.g., age, sex, prevalence of comorbidities, etc.). Statistical heterogeneity will be assessed using the variance estimates from the random effects model. It is likely that there will be significant heterogeneity between studies, and we will therefore pool results when studies are comparable. All analyses will be performed using STATA (version 16).

## Anticipated Results

Through this systematic review, we expect to identify the most common causes of acute pediatric critical illness resulting in hospital admission and mortality in LMICs by age and region. If data are available, we will also show temporal trends in admission and mortality by age and region. We will classify causes of admission as critical or non-critical and illustrate the global prevalence of critical illness with a map. Furthermore, we anticipate identifying diagnoses with the highest CFR for each age and region and illustrating these results through a series of forest plots for all-cause mortality, cause-specific mortality (pneumonia, sepsis, diarrhea, malaria), critical illness, and hospital length of stay (data permitting).

There are several advantages to the proposed approach. First, with broad inclusion criteria, we expect to capture most if not all relevant texts. Second, by not restricting the search to exclusively pediatric intensive care populations, we will be able to calculate the prevalence of critical illness across settings, including those without a formal PICU. Third, by including both individual LMICs by name and terms such as “resource-limited,” “low income,” and “developing” in the search strategy, we will likely identify more texts from LMICs, which will provide a more complete assessment of the burden of critical and non-critical disease in these countries.

There are potential limitations to the proposed protocol. First, neonatal and adolescent populations are included in some pediatric studies, and the search was not designed to capture these populations. We will intentionally exclude exclusively neonatal and adolescent populations from data analyses and will not be able to draw conclusions about children <28 days or >12 years of age. Second, we will exclude disease-specific studies that do not report overall pediatric hospital admissions, which may result in an underestimation of disease prevalence. Additionally, estimates will not include disease prevalence during outbreaks, potentially underestimating the true prevalence of disease and overall required critical care capacity. Third, we will restrict study inclusion to publications in Spanish, French, or English, and may not identify all potentially relevant texts. Fourth, we may underestimate the true burden of critical illness in LMICs by excluding emergency department or PICU population studies that lack the denominator of interest (general hospital admissions). However, without a common denominator, we cannot draw comparisons across studies. Sixth, it is possible that critical, but rare illnesses, will not be adequately represented in this systematic review as they are often categorized in the “other” category in texts. This systematic review will, however, describe the most common causes of pediatric critical illness, which is of greatest importance when the objective is to improve overall child health outcomes and inform resource allocation. Finally, we expect to include a small number of studies where the denominator does not represent the entire general pediatric admission population due to original study-imposed exclusions. The degree of bias from these texts should be minimal because only those with a similar case mix to the overall, general pediatric admission population will be included.

## Discussion

There is intense competition for limited resources in many LMICs and children are frequently overlooked as the global focus shifts away from infectious diseases toward non-communicable diseases, which are far more common in adult populations (23). To decrease childhood morbidity and mortality, health systems require capacity to deliver both preventative medicine and treatment, such as proven, effective therapies, like critical care (23). While dedicated PICUs are being developed in LMICs, clinician, and staff education is sub-optimal due to a lack of appreciation of the common pediatric critical illnesses.

The objective of this systematic review is to describe the most common causes of critical illnesses causing hospitalization and death in children in LMICs. This will provide much needed insight into the burden, etiology, and distribution of pediatric critical illness in LMICs, especially in settings where formal critical care services may not be currently available. Region-specific data that capture the burden of disease and outcomes for children in LMICs are essential to inform educational initiatives and training, shape advocacy and policy objectives, allocate limited resources appropriately, and implement context-appropriate, evidence-based critical care interventions for children in need. This systematic review is a crucial first step in setting future educational, advocacy, policy, research, and health delivery priorities for children with acute critical illness in LMICs.

## Data Availability Statement

The original contributions presented in the study are included in the article/Supplementary Material, further inquiries can be directed to the corresponding author/s.

## Author Contributions

TK: substantial contributions to the conception, design of the work, drafting, and revising the work. KN, RM, HR, NOB, JL, JA, EB, CB, YF, HJ, ML-L, ACM, AMM, MLd, SH, SS, NK, KR, CP, YT, MW, and AB: substantial contributions to the conception, design of the work, and revising the work critically for important intellectual content. All authors provided final approval of the version to be published and agree to be accountable for all aspects of the work.

## Funding

Research effort to create this publication was supported by the National Institute of Allergy and Infectious Diseases (award number K23AI144029, TK; award number 5U01AI126610, AB) of the National Institutes of Health (NIH), the Eunice Kennedy Shriver National Institute of Child Health and Human Development (award number 1R21HD106252-01, NO) of the NIH, the National Institute of General Medical Sciences (award number 5K08GM129763, KR) of the NIH, the National Medical Research Council, Singapore (MOH-000446-00, JL), the National Institute for Health Research (NIHR) Birmingham Biomedical Research Center of the National Health Services (YT), and Grand challenges Canada (NK). The views expressed are those of the author(s) and not necessarily those of the NIH, Singapore MOH, NHS, NIHR, or the Department of Health and Social Care.

## Conflict of Interest

The authors declare that the research was conducted in the absence of any commercial or financial relationships that could be construed as a potential conflict of interest. The reviewer YHM declared a shared affiliation with one of the authors JL to the handling editor at the time of review.

## Publisher's Note

All claims expressed in this article are solely those of the authors and do not necessarily represent those of their affiliated organizations, or those of the publisher, the editors and the reviewers. Any product that may be evaluated in this article, or claim that may be made by its manufacturer, is not guaranteed or endorsed by the publisher.
