# Supplementary material for: The Burden of Critical Illness in Hospitalized Children in Low- and Middle-Income Countries: Protocol for a Systematic Review and Meta-Analysis
Source: Front Pediatr. 2022 Mar 16;10:756643. doi: 10.3389/fped.2022.756643 (PMC8970052; doi:10.3389/fped.2022.756643)
Supplement: Supplementary file 1 [file Table_1.pdf]

Supplementary Material

Supplemental Table 1. Detailed search strategy by database

Database	Search Strategy
Ovid MEDLINE[#]	1. *hospitalization/ or *patient admission/ or *patient readmission/<br>2. (admis* or admit* or patient discharge* or readmis* or readmit* or rehospital*).tw,kf. or hospitali*.kf.<br>3. hospital mortality/ or ((tertiary or hospital*) adj5 (mortalit* or utilis* or utiliz*)).tw,kf.<br>4. (*hospital units/ or *hemodialysis units, hospital/ or *intensive care units/ or *intensive care units, pediatric/ or *respiratory care units/ or exp *hospitals/ or *tertiary care centers/) and mortalit*.mp.<br>5. (*critical care/ or early goal-directed therapy/ or (acute care or critical care or intensive care or iicu* or picu* or tertiary care).tw,kf.) and mortalit*.mp.<br>6. (child, hospitalized/ or hospitals, pediatric/) and mortalit*.mp.<br>7. or/1-6<br>8. (Afghanistan or Albania or Algeria or Samoa or Angola or Bangladesh or Belize or Benin or Bhutan or Bolivia or Botswana or Brazil or Burkina Faso or Burundi or Cabo Verde or Cambodia or Cameroon or Central African Republic or Chad or Timor or Cape Verde or Colombia or Comoros or Congo or Costa Rica or "Cote d'Ivoire" or Cuba or Djibouti or Dominica or Dominican Republic or Ecuador or Egypt or El Salvador or Equatorial Guinea or Eritrea or Ethiopia or Fiji or Gabon or Gambia or Ghana or Grenada or Guatemala or Guinea or Guinea-Bissau or Guyana or Haiti or Honduras or India or Indonesia or Iraq or Jamaica or Jordan or Kenya or Kiribati or North Korea or "Democratic People's Republic of Korea" or Kyrgyz Republic or Kyrgyzstan or Laos or "Lao People's Democratic Republic" or Lesotho or Liberia or Madagascar or Malawi or Maldives or Seychelles or Mali or Marshall Islands or Mexico or Micronesia or Moldova or Mongolia or Morocco or Mozambique or Myanmar or Namibia or Nepal or Nicaragua or Niger or Nigeria or Pakistan or Palestine or Panama or Papua New Guinea or Paraguay or Peru or Philippines or Rwanda or Sao Tome or Principe or Senegal or Sierra Leone or Solomon Islands or Somalia or South Africa or South Sudan or Sri Lanka or St Lucia or Saint Lucia or St Vincent or Saint Vincent or Grenadines or Sudan or Suriname or Swaziland or Syrian Arab Republic or Syria or Tajikistan or Tanzania or Thailand or Timor Leste or Togo or Tonga or Trinidad or Tobago or Tunisia or Turkmenistan or Uganda or Uzbekistan or Vanuatu or Venezuela or Vietnam or West Bank or Gaza or Yemen or Zambia or Zimbabwe or Mauritania).ti,ab,kf,sh. [LMIC title, abstract, author kw, MeSH]<br>9. Developing Countries/ or (developing adj1 (nation? or countr*)).tw,kf.<br>10. (resource* adj1 (constrain* or limit* or low* or poor* or restrict*)).tw. or (resource constrain* or resource limit*).kf.<br>11. ((low* or middl*) adj1 income countr*).tw. or (low income* or middle income* or LMIC).kf.<br>12. ((developing or least* or less* or limit* or third world or underdevelop* or under develop*) adj3 (countr* or nation* or setting*)).mp.<br>13. emerging econom*.mp.<br>14. ((low or middle) adj3 (socio demographic index or SDI)).mp.<br>15. or/8-14<br>16. infant/ or child, preschool/ or child/ or (pediatric* or paediatric* or child* or baby or babies or infan* or toddler* or preschool* or preteen* or pre teen* or preadolescen* or pre adolescen* or youth* or youngster* or boy* or girl* or juvenile*).tw,kf,so,jw<br>17. (comment or editorial or letter or news).pt.<br>18. (7 and 15 and 16) not 17<br>19. (201911* or 2019 11* or 201912* or 2019 12*).dp,dt,ed,ep,ez.<br>20. limit 18 to yr="2020 -Current"<br>21. (18 and 19) or 20
EMBASE	#17 #16 AND [1-11-2019]/sd NOT [2-3-2021]/sd

	#16 #6 AND #14 AND #15 NOT ('conference abstract'/it OR 'conference review'/it OR 'letter'/it OR 'editorial'/it OR 'note'/it)<br>#15 [infant]/lim OR [child]/lim OR [preschool]/lim OR [school]/lim OR pediatric*:ti,ab,kw OR paediatric*:ti,ab,kw OR child*:ti,ab,kw OR baby:ti,ab,kw OR babies:ti,ab,kw OR infan*:ti,ab,kw OR toddler*:ti,ab,kw OR preschool*:ti,ab,kw OR preteen*:ti,ab,kw OR preadolescen*:ti,ab,kw OR ((pre NEXT/1 (adolescen* OR teen*)):ti,ab,kw) OR youth*:ti,ab,kw OR youngster*:ti,ab,kw OR boy*:ti,ab,kw OR girl*:ti,ab,kw OR juvenile*:ti,ab,kw<br>#14 #7 OR #8 OR #9 OR #10 OR #11 OR #12 OR #13<br>#13 (('low middle' OR middle) NEAR/3 ('socio demographic index' OR sdi)):ti,ab,kw,de<br>#12 'emerging economy':ti,ab,kw,de OR 'emerging economies':ti,ab,kw,de<br>#11 ((developing OR underdevelop* OR underdeveloped OR least OR less OR limit* OR 'third world') NEAR/3 (countr* OR nation* OR setting*)):ti,ab,kw,de<br>#10 (((low* OR middl*) NEAR/2 countr*):ti,ab,kw) OR 'low income':ti,ab,kw OR 'middle income':ti,ab,kw OR lmic:ti,ab,kw<br>#9 (resource* NEAR/1 (constrain* OR limit* OR low* OR poor* OR restrict*)):ti,ab,kw<br>#8 'developing country'/exp OR ((developing NEAR/1 (nation* OR countr*)):ti,ab,kw)<br>#7 'afghanistan':ti,ab,de,kw OR 'albania':ti,ab,de,kw OR 'algeria':ti,ab,de,kw OR 'samoa':ti,ab,de,kw OR 'angola':ti,ab,de,kw OR 'bangladesh':ti,ab,de,kw OR 'belize':ti,ab,de,kw OR 'benin':ti,ab,de,kw OR 'bhutan':ti,ab,de,kw OR 'bolivia':ti,ab,de,kw OR 'botswana':ti,ab,de,kw OR 'brazil':ti,ab,de,kw OR 'burkina faso':ti,ab,de,kw OR 'burundi':ti,ab,de,kw OR 'cabo verde':ti,ab,de,kw OR 'cambodia':ti,ab,de,kw OR 'cameroon':ti,ab,de,kw OR 'central african republic':ti,ab,de,kw OR 'chad':ti,ab,de,kw OR 'timor':ti,ab,de,kw OR 'cape verde':ti,ab,de,kw OR 'colombia':ti,ab,de,kw OR 'comoros':ti,ab,de,kw OR 'congo':ti,ab,de,kw OR 'costa rica':ti,ab,de,kw OR 'cote d ivoire':ti,ab,de,kw OR 'cuba':ti,ab,de,kw OR 'djibouti':ti,ab,de,kw OR 'dominica':ti,ab,de,kw OR 'dominican republic':ti,ab,de,kw OR 'ecuador':ti,ab,de,kw OR 'egypt':ti,ab,de,kw OR 'el salvador':ti,ab,de,kw OR 'equatorial guinea':ti,ab,de,kw OR 'eritrea':ti,ab,de,kw OR 'ethiopia':ti,ab,de,kw OR 'fiji':ti,ab,de,kw OR 'gabon':ti,ab,de,kw OR 'gambia':ti,ab,de,kw OR 'ghana':ti,ab,de,kw OR 'grenada':ti,ab,de,kw OR 'guatemala':ti,ab,de,kw OR 'guinea':ti,ab,de,kw OR 'guinea bissau':ti,ab,de,kw OR 'guyana':ti,ab,de,kw OR 'haiti':ti,ab,de,kw OR 'honduras':ti,ab,de,kw OR 'india':ti,ab,de,kw OR 'indonesia':ti,ab,de,kw OR 'iraq':ti,ab,de,kw OR 'jamaica':ti,ab,de,kw OR 'jordan':ti,ab,de,kw OR 'kenya':ti,ab,de,kw OR 'kiribati':ti,ab,de,kw OR 'north korea':ti,ab,de,kw OR 'democratic people s republic of korea':ti,ab,de,kw OR 'kyrgyz republic':ti,ab,de,kw OR 'kyrgyzstan':ti,ab,de,kw OR 'laos':ti,ab,de,kw OR 'lao people s democratic republic':ti,ab,de,kw OR 'lesotho':ti,ab,de,kw OR 'liberia':ti,ab,de,kw OR 'madagascar':ti,ab,de,kw OR 'malawi':ti,ab,de,kw OR 'maldives':ti,ab,de,kw OR 'seychelles':ti,ab,de,kw OR 'mali':ti,ab,de,kw OR 'marshall islands':ti,ab,de,kw OR 'mexico':ti,ab,de,kw OR 'micronesia':ti,ab,de,kw OR 'moldova':ti,ab,de,kw OR 'mongolia':ti,ab,de,kw OR 'morocco':ti,ab,de,kw OR 'mozambique':ti,ab,de,kw OR 'myanmar':ti,ab,de,kw OR 'namibia':ti,ab,de,kw OR 'nepal':ti,ab,de,kw OR 'nicaragua':ti,ab,de,kw OR 'niger':ti,ab,de,kw OR 'nigeria':ti,ab,de,kw OR 'pakistan':ti,ab,de,kw OR 'palestine':ti,ab,de,kw OR 'panama':ti,ab,de,kw OR 'papua new guinea':ti,ab,de,kw OR 'paraguay':ti,ab,de,kw OR 'peru':ti,ab,de,kw OR 'philippines':ti,ab,de,kw OR 'rwanda':ti,ab,de,kw OR 'sao tome':ti,ab,de,kw OR 'principe':ti,ab,de,kw OR 'senegal':ti,ab,de,kw OR 'sierra leone':ti,ab,de,kw OR 'solomon islands':ti,ab,de,kw OR 'somalia':ti,ab,de,kw OR 'south africa':ti,ab,de,kw OR 'south sudan':ti,ab,de,kw OR 'sri lanka':ti,ab,de,kw OR 'st lucia':ti,ab,de,kw OR 'saint lucia':ti,ab,de,kw OR 'st vincent':ti,ab,de,kw OR 'saint vincent':ti,ab,de,kw OR 'grenadines':ti,ab,de,kw OR 'sudan':ti,ab,de,kw OR 'suriname':ti,ab,de,kw OR 'swaziland':ti,ab,de,kw OR 'syrian arab republic':ti,ab,de,kw OR 'syria':ti,ab,de,kw OR 'tajikistan':ti,ab,de,kw OR 'tanzania':ti,ab,de,kw OR 'thailand':ti,ab,de,kw OR 'timor leste':ti,ab,de,kw OR 'togo':ti,ab,de,kw OR 'tonga':ti,ab,de,kw OR 'trinidad':ti,ab,de,kw OR 'tobago':ti,ab,de,kw OR 'tunisia':ti,ab,de,kw OR 'turkmenistan':ti,ab,de,kw OR 'uganda':ti,ab,de,kw OR 'uzbekistan':ti,ab,de,kw OR 'vanuatu':ti,ab,de,kw OR 'venezuela':ti,ab,de,kw OR 'vietnam':ti,ab,de,kw OR 'west bank':ti,ab,de,kw OR 'gaza':ti,ab,de,kw OR 'yemen':ti,ab,de,kw OR 'zambia':ti,ab,de,kw OR 'zimbabwe':ti,ab,de,kw OR 'mauritania':ti,ab,de,kw

	#6 #1 OR #2 OR #3 OR #4 OR #5<br>#5 ((((acute OR critical OR intensive) NEXT/1 care):ti,ab) OR (((hemodialysis OR respiratory) NEXT/2 (unit* OR ward*)):ti,ab) OR iicu*:ti,ab OR picu*:ti,ab) AND mortalit*<br>#4 ('intensive care unit'/mj OR 'medical intensive care unit'/mj OR 'pediatric intensive care unit'/mj OR 'hospital'/exp/mj OR 'tertiary care center'/mj OR 'intensive care'/exp/mj OR 'hospital patient'/mj OR 'hospitalized child'/mj) AND mortalit*<br>#3 'hospital mortality'/mj OR (((tertiary OR hospital*) NEAR/5 (mortalit* OR utilis* OR utiliz*)):ti,ab)<br>#2 admis*:ti,ab OR admit*:ti,ab OR ((patient NEXT/1 discharge*):ti,ab) OR readmis*:ti,ab OR readmit*:ti,ab OR rehospital*:ti,ab<br>#1 'hospitalization'/mj OR 'hospital admission'/mj OR 'hospital readmission'/mj
CINAHL	S21 S18 OR S20<br>S20 S7 AND S17 AND S19 AND Limiters - Published Date: 20191101-20211231<br>S19 TI (pediatric* OR paediatric* OR child* OR baby OR babies OR infan* OR toddler* OR preteen* OR (pre W1 teen*) OR preadolescen* OR (pre W1 adolescen*) OR youth* OR youngster* OR boy* OR girl* OR juvenile*) OR AB (pediatric* OR paediatric* OR child* OR baby OR babies OR infan* OR toddler* OR preschool* OR preteen* OR (pre W1 teen*) OR preadolescen* OR (pre W1 adolescen*) OR youth* OR youngster* OR boy* OR girl* OR juvenile*) OR JN (pediatric* OR paediatric* OR child* OR baby OR babies OR infan* OR toddler* OR preteen* OR (pre W1 teen*) OR preadolescen* OR (pre W1 adolescen*) OR youth* OR youngster* OR boy* OR girl* OR juvenile*)<br>S18 S7 AND S17 Limiters - Age Groups: Infant: 1-23 months, Child, Preschool: 2-5 years, Child: 6-12 years; Published Date: 20191101-20211231<br>S17 S8 OR S9 OR S10 OR S11 OR S12 OR S13 OR S14 OR S15 OR S16<br>S16 TI ((low OR middl*) N3 (socio demographic index OR sdi)) OR AB ((low OR middl*) N3 (socio demographic index OR sdi))<br>S15 TI (emerging N1 econom*) OR AB (emerging N1 econom*)<br>S14 TI ((developing OR least* OR less* OR limit* OR "third world" OR underdevelop* OR (under W1 develop*)) N3 (countr* OR nation* OR setting*)) OR AB ((developing OR least* OR less* OR limit* OR "third world" OR underdevelop* OR (under W1 develop*)) N3 (countr* OR nation* OR setting*))<br>S13 TI ((low* OR middl*) N1 income countr*) OR AB ((low* OR middl*) N1 income countr*) OR TI LMIC OR AB LMIC<br>S12 TI (resource* N1 (constrain* OR limit* OR low* OR poor* OR restrict*)) OR AB (resource* N1 (constrain* OR limit* OR low* OR poor* OR restrict*))<br>S11 (MH "Developing Countries") OR TI (developing N1 (nation* or countr*)) OR AB (developing N1 (nation* or countr*))<br>S10 MW Afghanistan or Albania or Algeria or Samoa or Angola or Bangladesh or Belize or Benin or Bhutan or Bolivia or Botswana or Brazil or “Burkina Faso” or Burundi or “Cabo Verde” or Cambodia or Cameroon or “Central African Republic” or Chad or Timor or “Cape Verde” or Colombia or Comoros or Congo or “Costa Rica” or “Cote d'Ivoire” or Cuba or Djibouti or Dominica or “Dominican Republic” or Ecuador or Egypt or “El Salvador” or “Equatorial Guinea” or Eritrea or Ethiopia or Fiji or Gabon or Gambia or Ghana or Grenada or Guatemala or Guinea or “Guinea-Bissau” or Guyana or Haiti or Honduras or India or Indonesia or Iraq or Jamaica or Jordan or Kenya or Kiribati or “North Korea” or Korea or “Kyrgyz Republic” or Kyrgyzstan or Laos or "Lao People's Democratic Republic" or Lesotho or Liberia or Madagascar or Malawi or Maldives or Seychelles or Mali or “Marshall Islands” or Mexico or Micronesia or Moldova or Mongolia or Morocco or Mozambique or Myanmar or Namibia or Nepal or Nicaragua or Niger or Nigeria or Pakistan or Palestine or Panama or “Papua New Guinea” or Paraguay or Peru or Philippines or Rwanda or “Sao Tome” or “Principe or Senegal” or “Sierra Leone” or “Solomon Islands” or Somalia or “South Africa” or “South Sudan” or “Sri Lanka” or “St Lucia” or “Saint Lucia” or “St Vincent” or “Saint Vincent” or Grenadines or Sudan or Suriname or Swaziland or “Syrian Arab Republic” or Syria or Tajikistan or Tanzania or Thailand or “Timor Leste” or Togo or Tonga or Trinidad or Tobago or Tunisia or Turkmenistan or Uganda or Uzbekistan or Vanuatu or Venezuela or Vietnam or “West Bank” or Gaza or Yemen or Zambia or Zimbabwe or Mauritania<br>S9 AB Afghanistan or Albania or Algeria or Samoa or Angola or Bangladesh or Belize or Benin or Bhutan or Bolivia or Botswana or Brazil or “Burkina Faso” or Burundi or “Cabo Verde” or

	Cambodia or Cameroon or “Central African Republic” or Chad or Timor or “Cape Verde” or Colombia or Comoros or Congo or “Costa Rica” or “Cote d'Ivoire” or Cuba or Djibouti or Dominica or “Dominican Republic” or Ecuador or Egypt or “El Salvador” or “Equatorial Guinea” or Eritrea or Ethiopia or Fiji or Gabon or Gambia or Ghana or Grenada or Guatemala or Guinea or “Guinea-Bissau” or Guyana or Haiti or Honduras or India or Indonesia or Iraq or Jamaica or Jordan or Kenya or Kiribati or “North Korea” or Korea or “Kyrgyz Republic” or Kyrgyzstan or Laos or "Lao People's Democratic Republic" or Lesotho or Liberia or Madagascar or Malawi or Maldives or Seychelles or Mali or “Marshall Islands” or Mexico or Micronesia or Moldova or Mongolia or Morocco or Mozambique or Myanmar or Namibia or Nepal or Nicaragua or Niger or Nigeria or Pakistan or Palestine or Panama or “Papua New Guinea” or Paraguay or Peru or Philippines or Rwanda or “Sao Tome” or “Principe or Senegal” or “Sierra Leone” or “Solomon Islands” or Somalia or “South Africa” or “South Sudan” or “Sri Lanka” or “St Lucia” or “Saint Lucia” or “St Vincent” or “Saint Vincent” or Grenadines or Sudan or Suriname or Swaziland or “Syrian Arab Republic” or Syria or Tajikistan or Tanzania or Thailand or “Timor Leste” or Togo or Tonga or Trinidad or Tobago or Tunisia or Turkmenistan or Uganda or Uzbekistan or Vanuatu or Venezuela or Vietnam or “West Bank” or Gaza or Yemen or Zambia or Zimbabwe or Mauritania<br>S8 TI Afghanistan or Albania or Algeria or Samoa or Angola or Bangladesh or Belize or Benin or Bhutan or Bolivia or Botswana or Brazil or “Burkina Faso” or Burundi or “Cabo Verde” or Cambodia or Cameroon or “Central African Republic” or Chad or Timor or “Cape Verde” or Colombia or Comoros or Congo or “Costa Rica” or “Cote d'Ivoire” or Cuba or Djibouti or Dominica or “Dominican Republic” or Ecuador or Egypt or “El Salvador” or “Equatorial Guinea” or Eritrea or Ethiopia or Fiji or Gabon or Gambia or Ghana or Grenada or Guatemala or Guinea or “Guinea-Bissau” or Guyana or Haiti or Honduras or India or Indonesia or Iraq or Jamaica or Jordan or Kenya or Kiribati or “North Korea” or Korea or “Kyrgyz Republic” or Kyrgyzstan or Laos or "Lao People's Democratic Republic" or Lesotho or Liberia or Madagascar or Malawi or Maldives or Seychelles or Mali or “Marshall Islands” or Mexico or Micronesia or Moldova or Mongolia or Morocco or Mozambique or Myanmar or Namibia or Nepal or Nicaragua or Niger or Nigeria or Pakistan or Palestine or Panama or “Papua New Guinea” or Paraguay or Peru or Philippines or Rwanda or “Sao Tome” or “Principe or Senegal” or “Sierra Leone” or “Solomon Islands” or Somalia or “South Africa” or “South Sudan” or “Sri Lanka” or “St Lucia” or “Saint Lucia” or “St Vincent” or “Saint Vincent” or Grenadines or Sudan or Suriname or Swaziland or “Syrian Arab Republic” or Syria or Tajikistan or Tanzania or Thailand or “Timor Leste” or Togo or Tonga or Trinidad or Tobago or Tunisia or Turkmenistan or Uganda or Uzbekistan or Vanuatu or Venezuela or Vietnam or “West Bank” or Gaza or Yemen or Zambia or Zimbabwe or Mauritania<br>S7 S1 OR S2 OR S3 OR S4 OR S5 OR S6<br>S6 ((MH "Critical Care") OR (MH "Hospitals, Pediatric") OR (MH "Child, Hospitalized")) AND TX mortalit*<br>S5 (TI (("acute care" OR "critical care" OR "intensive care" OR iicu* OR picu* OR "tertiary care") AND TX mortalit*) OR (AB (("acute care" OR "critical care" OR "intensive care" OR iicu* OR picu* OR "tertiary care") AND TX mortalit*)<br>S4 ((MH "Hospital Units") OR (MH "Pediatric Units") OR (MH "Intensive Care Units") OR (MH "Intensive Care Units, Pediatric") OR (MH "Respiratory Care Units") OR (MH "Tertiary Health Care")) AND TX mortalit<br>S3 (MH "Hospital Mortality") OR (TI ((tertiary OR hospital*) N5 (mortalit* OR utilis* OR utiliz*))) OR (AB ((tertiary OR hospital*) N5 (mortalit* OR utilis* OR utiliz*)))<br>S2 TI (admis* OR admit* OR (patient W1 discharge*) OR readmis* OR readmit* OR rehospital*) OR AB (admis* OR admit* OR (patient W1 discharge*) OR readmis* OR readmit* OR rehospital*)<br>S1 (MH "Hospitalization") OR (MH "Patient Admission") OR (MH "Readmission")
LILACS	VHL Advanced Search<br>#1<br>(mh:("Hospitalization" OR "Child, Hospitalized" OR "Patient Admission" OR "Patient Readmission" OR "Hospital Mortality")) AND (tw:(develop* OR desarrollo OR resource* OR "third world" OR underdeveloped OR under developed OR low income OR bajos ingresos OR

	middle income OR ingreso medio OR LMIC OR emerging econom* OR "socio demographic index" OR SDI OR Afghanistan OR Albania OR Algeria OR Samoa OR Angola OR Bangladesh OR Belize OR Benin OR Bhutan OR Bolivia OR Botswana OR Brazil OR "Burkina Faso" OR Burundi OR "Cabo Verde" OR Cambodia OR Cameroon OR "Central African Republic" OR Chad OR Timor OR "Cape Verde" OR Colombia OR Comoros OR Congo OR "Costa Rica" OR "Cote d'Ivoire" OR Cuba OR Djibouti OR Dominica OR "Dominican Republic" OR Ecuador OR Egypt OR "El Salvador" OR "Equatorial Guinea" OR Eritrea OR Ethiopia OR Fiji OR Gabon OR Gambia OR Ghana OR Grenada OR Guatemala OR Guinea OR "Guinea-Bissau" OR Guyana OR Haiti OR Honduras OR India OR Indonesia OR Iraq OR Jamaica OR Jordan OR Kenya OR Kiribati OR "North Korea" OR Korea OR "Kyrgyz Republic" OR Kyrgyzstan OR Laos OR "Lao People's Democratic Republic" OR Lesotho OR Liberia OR Madagascar OR Malawi OR Maldives OR Seychelles OR Mali OR "Marshall Islands" OR Mexico OR Micronesia OR Moldova OR Mongolia OR Morocco OR Mozambique OR Myanmar OR Namibia OR Nepal OR Nicaragua OR Niger OR Nigeria OR Pakistan OR Palestine OR Panama OR "Papua New Guinea" OR Paraguay OR Peru OR Philippines OR Rwanda OR "Sao Tome" OR "Principe or Senegal" OR "Sierra Leone" OR "Solomon Islands" OR Somalia OR "South Africa" OR "South Sudan" OR "Sri Lanka" OR "St Lucia" OR "Saint Lucia" OR "St Vincent" OR "Saint Vincent" OR Grenadines OR Sudan OR Suriname OR Swaziland OR "Syrian Arab Republic" OR Syria OR Tajikistan OR Tanzania OR Thailand OR "Timor Leste" OR Togo OR Tonga OR Trinidad OR Tobago OR Tunisia OR Turkmenistan OR Uganda OR Uzbekistan OR Vanuatu OR Venezuela OR Vietnam OR "West Bank" OR Gaza OR Yemen OR Zambia OR Zimbabwe OR Mauritania)) AND (tw:(pediatric* OR paediatric* OR child* OR baby OR babies OR infan* OR toddler* OR preschool* OR preteen* OR preadolescen* OR youth* OR youngster* OR boy* OR girl* OR juvenil* OR nina OR nino OR ninit* OR preescholar* OR chico* OR chica*))<br>Limited To: LILACS and 2019-2021<br><br>#2<br>(tw:(critical care OR cuidados críticos OR intensive care OR respiratory care OR cuidados respiratorios OR hospital* OR tertiary OR terciaria)) AND (tw:(mortali* OR admis* OR admit* OR readmis* OR readmit* OR rehospital*)) AND (tw:(develop* OR desarrollo OR resource* OR "third world" OR underdeveloped OR under developed OR low income OR bajos ingresos OR middle income OR ingreso medio OR LMIC OR emerging econom* OR "socio demographic index" OR SDI OR Afghanistan OR Albania OR Algeria OR Samoa OR Angola OR Bangladesh OR Belize OR Benin OR Bhutan OR Bolivia OR Botswana OR Brazil OR "Burkina Faso" OR Burundi OR "Cabo Verde" OR Cambodia OR Cameroon OR "Central African Republic" OR Chad OR Timor OR "Cape Verde" OR Colombia OR Comoros OR Congo OR "Costa Rica" OR "Cote d'Ivoire" OR Cuba OR Djibouti OR Dominica OR "Dominican Republic" OR Ecuador OR Egypt OR "El Salvador" OR "Equatorial Guinea" OR Eritrea OR Ethiopia OR Fiji OR Gabon OR Gambia OR Ghana OR Grenada OR Guatemala OR Guinea OR "Guinea-Bissau" OR Guyana OR Haiti OR Honduras OR India OR Indonesia OR Iraq OR Jamaica OR Jordan OR Kenya OR Kiribati OR "North Korea" OR Korea OR "Kyrgyz Republic" OR Kyrgyzstan OR Laos OR "Lao People's Democratic Republic" OR Lesotho OR Liberia OR Madagascar OR Malawi OR Maldives OR Seychelles OR Mali OR "Marshall Islands" OR Mexico OR Micronesia OR Moldova OR Mongolia OR Morocco OR Mozambique OR Myanmar OR Namibia OR Nepal OR Nicaragua OR Niger OR Nigeria OR Pakistan OR Palestine OR Panama OR "Papua New Guinea" OR Paraguay OR Peru OR Philippines OR Rwanda OR "Sao Tome" OR "Principe or Senegal" OR "Sierra Leone" OR "Solomon Islands" OR Somalia OR "South Africa" OR "South Sudan" OR "Sri Lanka" OR "St Lucia" OR "Saint Lucia" OR "St Vincent" OR "Saint Vincent" OR Grenadines OR Sudan OR Suriname OR Swaziland OR "Syrian Arab Republic" OR Syria OR Tajikistan OR Tanzania OR Thailand OR "Timor Leste" OR Togo OR Tonga OR Trinidad OR Tobago OR Tunisia OR Turkmenistan OR Uganda OR Uzbekistan OR Vanuatu OR Venezuela OR Vietnam OR "West Bank" OR Gaza OR Yemen OR Zambia OR Zimbabwe OR Mauritania)) AND (tw:(pediatric* OR paediatric* OR child* OR baby OR babies OR infan* OR toddler* OR preschool* OR preteen* OR preadolescen* OR youth* OR youngster* OR boy* OR girl* OR juvenil* OR nina OR nino OR ninit* OR preescholar* OR chico* OR chica*))
[#] MEDLINE strategy abbreviations:	

ab = abstract
jw = journal word
kf = keyword heading word
mp = title, abstract, original title, name of substance word, subject heading word, floating sub-heading word, keyword heading word, organism supplementary concept word, protocol supplementary concept word, rare disease supplementary concept word, unique identifier, synonyms
pt = publication type
sh = MeSH subject heading
so = source
ti = title
tw = text word in abstract or title
